# Correcting near vision impairment and women’s empowerment: a before-after mixed-methods study among older Zanzibari craftswomen

**DOI:** 10.1136/bmjopen-2024-086624

**Published:** 2024-11-14

**Authors:** Fatma Omar, Omar Juma Othman, Ai Chee Yong, Emma McConnell, Damaris Mulewa, Christine Graham, Kajal Shah, Michelle Fernandes Martins, Adrianna Farmer, Ronnie Graham, Eden Mashayo, Ving Fai Chan

**Affiliations:** 1 Zanzibar Ministry of Health, Zanzibar, Mkoa wa Unguja Mjini Magh, Tanzania; 2 Centre for Public Health, Queen's University Belfast, Belfast, UK; 3 Independent Researcher, Nairobi, Kenya; 4 Dublin Institute of Technology, Dublin, Ireland; 5 Independent Researcher, Edinburgh, UK; 6 Vision Care Foundation, Dar-es-Salaam, Tanzania

**Keywords:** Aging, Health, Ophthalmology, Public health, QUALITATIVE RESEARCH

## Abstract

**Abstract:**

**Objective:**

To understand if presbyopia correction could empower older craftswomen entrepreneurs living in Zanzibar.

**Design:**

Mixed-method, before-after intervention study.

**Setting:**

Unguja and Pemba islands, Zanzibar.

**Participants:**

209 craftswomen (weaving, tailoring and sewing, pottery and producing oil and making soaps) 40 years and older with correctable presbyopia, with no other ocular morbidities.

**Intervention:**

Eye health assessment and near vision spectacle correction for 6 months.

**Primary and secondary outcomes:**

Change in economic, social, psychological and political empowerment (4-point Likert scale responses) before and after correction. Odds ratios were calculated to determine the likelihood of upward movement on the Likert scale. Five focus group interviews were conducted to explore the craftswomen’s daily experiences concerning vision correction and empowerment, and subsequently, narrative analysis was conducted.

**Results:**

Of the 209 craftswomen who completed the baseline survey from 4 to 21 April 2022, 157 (75.1%) were successfully followed up from 6 to 27 October 2022. Craftswomen reported significantly greater economic, social, psychological and political empowerment in 14 out of 18 statements (77.8%) (p<0.05). Qualitative responses showed that after correction, craftswomen reported having greater autonomy in running the business and improved income, better decision-making power for their business and children, greater independence and confidence and greater participation in problem-solving for the community and appointing leaders. We did not observe a significant change in the following: ability to make decisions for their family, understanding their capabilities, ability to be elected as a leader and ability to advise government leaders.

**Conclusion:**

The correlation between presbyopia correction and empowerment among older Zanzibari craftswomen is mostly positive. Some aspects of empowerment require further investigation with an extended timeframe.

**STRENGTHS AND LIMITATIONS OF THIS STUDY**

- The use of mixed-method design provides a comprehensive understanding from the association between presbyopic correction and empowerment among craftswomen in a lower-middle income country.
- The findings from this study are being used to inform government policy on providing eye care to older craftswomen in Zanzibar.
- The sample’s unique characteristics limit the result’s generalisability to other work settings.
- A non-trial study design makes it impossible to determine a causal relationship.
- Self-reported and positive phrasing questionnaire in this study may have introduced respondent bias and overestimated the intervention effects, and a short follow-up period may also underestimate or overestimate the intervention’s intended effects.

## Introduction

 Presbyopia, a natural age-related decline in the ability to focus on near objects, starts at 35 years old and affects almost everyone over 40 years old and can be easily corrected with a pair of near vision spectacles. In 2018, approximately 1.09 billion people worldwide lived with functional presbyopia,^1^ 826 million of whom are uncorrected or undercorrected.^2^ Low- and middle-income countries (LMICs) bear 90% of this burden,^3^ but the correction rate is as low as 10%.^4 5^ Substantial evidence^6^,^9^ has shown that uncorrected presbyopia can reduce work performance at close distances. Furthermore, one recent cohort study of female textile workers in Bangladesh showed that uncorrected presbyopia is associated with earning $6.51 less per month than those with correction and no vision loss.^10^ It has been estimated that the significant economic implication of not correcting presbyopia is equivalent to a total global productivity loss of US$25 billion.^11^ Trial^12^ and non-trial data^13^,^16^ have shown that correcting presbyopia could improve work productivity and quality of life^17 18^ substantially.

Uncorrected refractive error has been identified as one of the key reasons for preventable vision loss globally, typically affecting LMICs.^19^ It was also estimated that in 2020, of those who were visually impaired from uncorrected refractive error, 55% were women and girls.^19^ In 2021, the United Nations General Assembly adopted the ‘Vision for Everyone: Accelerating Action to Achieve the Sustainable Development Goals’ and committed to global eye health initiatives to address the current 1.1 billion people living with preventable sight loss by 2030. By working towards achieving these objectives, several Sustainable Development Goals (SDGs) will be addressed, namely, No Poverty (SDG 1), Good Health and Well-being (SDG 3), Gender Equality (SDG 5) and Decent Work and Economic Growth (SDG 8).

No work has been done to show if presbyopia correction could lead to women’s empowerment. Women’s empowerment aims to increase women’s sense of self-worth, decision-making autonomy and the right to make changes for themselves and those around them. Uncorrected presbyopia disproportionately affects 11% more women than men,^20^ with women facing greater financial, social and cultural barriers to eye health services^19^ and having 7.8% higher representation in informal and vulnerable employment.^21^ The barriers women in low-income countries face in different aspects of their lives could make it more challenging for them to achieve empowerment.

Women in Zanzibar face situations like those in many LMICs. Women comprise more than half of the population (1.62 million), a third are of working age and a quarter head a household.^21^ The Zanzibari population is also predominantly Muslim, with well-defined gender roles where men are considered the household providers/leaders; women are expected to be submissive and obey their husbands.^22^ Women have poorer participation in education and employment than men, leading to about 9.3% of women who head households in urban areas and 3.4% in rural areas working in the craft industry.^21^

In October 2021, the Women’s Empowerment through Investing in Zanzibari Craftswomen’s Eyesight (WE-ZACE) programme was initiated. The programme aimed to explore the craftswomen’s perception of empowerment (phase 1), validate presbyopia burden and understand attitudes to near vision correction (phase 2), understand vision and women’s empowerment, develop the Theory of Change and design the WE-ZACE programme (phase 3), provision of presbyopic correction and understand its effect on empowerment (phase 4) and reflection and listening workshop to identifying other interventions needed to foster social change (phase 5).

Our work found that only 2 of the 231 craftswomen with presbyopia were corrected adequately (presenting near vision equal to or better than N8 at 40 cm).^22^ The same cohort also perceived that their near vision spectacle correction could lead to different aspects of women’s empowerment from a personal and relational level, which include economic, social, psychological, educational and political empowerment.^23^ It was identified that older craftswomen perceived that correction of a near vision impairment caused by presbyopia could lead to far-reaching benefits in improved social connection, less reliance on others, greater financial stability, empowerment of other women, and greater independence and self-confidence.^23^

This paper aimed to answer, ‘*What is the effect of presbyopia correction on older Zanzibari craftswomen’s self-reported empowerment six months post-correction?’* (phase 4). We hypothesised that presbyopia correction could significantly improve self-reported empowerment. We also aimed to understand the women’s experiences that could explain the significant or insignificant empowerment observed.

## Methods

The study consisted of a 6-month pre-intervention and post-intervention quantitative component and a qualitative interpretive component, allowing for data triangulation. All participants provided informed consent, and all methods were carried out following the Declaration of Helsinki.

### Patient and public involvement

Craftswomen and local stakeholders were involved in the development of the eye programme through four patient and public involvement meetings during the initiation and development stage. They provided input on the research questions, suggestions on timing for recruitment and implementation. Craftswomen and local stakeholders were invited to the dissemination of the findings, with a breakout session to obtain their views and suggestions.

### Sampling, sample and recruitment

This study involved craftswomen 40 years and older in Zanzibar. Ophthalmic clinical officers and optometrists were responsible for conducting all eye examinations. Tumbling E Snellen charts were used to measure distance visual acuity (VA) at 6 m. If an individual’s vision was worse than 6/12 in either eye, they were administered an eye examination to rule out the presence of eye morbidities. Those with no eye morbidities were given a test to measure their refractive error. If their distance vision was 6/12 or better or they had a correctable refractive error, they were administered a near vision test. If their near vision was worse than N8 at 40 cm, they were considered presbyopic and provided a pair of free near vision spectacles (per national guidelines). To participate, they must have presenting or correctable distance VA 6/12 or better in both eyes, be presbyopic (unable to read the N8 line without glasses, but correctable with reading glasses) and not wear near vision spectacles or have poor near vision that could not be improved with spectacles. Those with distance refractive errors were corrected with a pair of free spectacles, and those with worse distance vision caused by other non-refractive errors were excluded. Nineteen women’s cooperatives from the two main islands of Zanzibar (Unguja and Pemba) took part, with a finite sampling representing all eligible craftswomen in the area.

Given the nature of a pilot and participatory action research, post hoc power calculations are more practical and informative because of a lack of prior information on women’s empowerment (key parameters). Based on our final sample size of 157, with a type II error rate (two-tailed)=5.0%, estimated effect size=0.15 and a SD=0.5, the power of the study reached 95%.

### Questionnaires and quantitative data analysis

Our outcome of interest was empowerment, a multifaceted concept typically measured through proxy indicators.^24^ In our context, we sought to provide the craftswomen with vision correction leading to empowerment that engages critical consciousness, emphasising the craftswomen’s autonomy as women, human beings and productive citizens.^25^ Therefore, consensus was reached among the research team and local stakeholders to measure women’s empowerment based on the craftswomen’s perception of ‘empowered women’ in their local context. To achieve this goal, we first developed a draft questionnaire of 31 items through qualitative responses from the craftswomen in an engagement workshop and semistructured interviews with 34 craftswomen from 15 to 18 October 2021. This draft included five domains: economic (5 statements), social (10 statements), education (4 statements), psychological (7 statements) and political (5 statements). We then surveyed a new cohort of 20 craftswomen on 1 March 2022 to determine the relevance of each statement (0=no relevance, 1=little relevance, 2=relevant, 3=highly relevant). Only statements with an average relevance score of 2 or more were included in the factor analysis to finalise the questionnaire. Due to the small sample size, only those with a cut-off of 0.7 or more are loaded into the factor analysis (online supplemental material 1). The final questionnaire consisted of four domains: economic (five statements), social (four statements), psychological (five statements) and political (four statements) (online supplemental material 2).

Before the craftswomen’s near vision was corrected, field workers administered a baseline survey verbally from 4 to 21 April 2022. The survey had two sections. Section one collected demographic data such as age, education, craft type, years of experience, marital status and number of dependents. Section two aimed to measure the four domains of empowerment using a 4-point Likert scale, with 1 being strongly disagree, 2 disagree, 3 agree and 4 strongly agree. Six months later, a follow-up survey was conducted from 6 to 27 October 2022. Given that many participants reported high levels of empowerment before the intervention, we assess the relative improvement in responses by calculating the ORs for each statement, focusing on the likelihood of upward movement on the Likert scale. Wilcoxon’s sign-rank test was used to determine the significance of the increase in the proportion of positive responses from pre-intervention to post-intervention (significance level at 5%).

### Focus group interviews and qualitative data analysis

In this qualitative component, 18 women were purposively selected to participate in three focus group interviews (five to eight participants per group). The sample size was determined based on the principles of data saturation, where the aim is to gather sufficient depth and diversity of experiences rather than to achieve a statistically representative sample. The participants were chosen to ensure variation across different crafts—weaving (n=5), tailoring and sewing (n=5), pottery (n=4), and producing oil and making soaps (n=4)—as well as geographical location (Unguja n=10, Pemba n=8) and experience level (less than 10 years, n=11; more than 10 years, n=7). This approach ensured a rich exploration of the craftswomen’s daily experiences concerning vision correction and empowerment. Data analysis was conducted concurrently with the focus group interviews, and by the third focus group, no new themes or significant insights were emerging. As the same themes—related to vision correction, different aspects of empowerment and craft practices—were consistently repeated across all groups, it became clear that saturation had been reached. Therefore, further data collection was deemed unlikely to yield additional novel information, confirming that the sample size was appropriate for this study.

We used indirect questioning and narrative methods (online supplemental material 3), including overarching questions and probes on financial, social, psychological and political aspects, to navigate the cultural sensitivities around the sensitive topic of empowerment in a patriarchal society. This approach minimises bias by encouraging more natural responses, as direct questioning might lead craftswomen to provide socially acceptable answers. Additionally, since empowerment is a relatively new concept for older women in Zanzibar, indirect questioning helps them express their thoughts more comfortably and accurately. Two Swahili-speaking field researchers from Zanzibar (FO, female, ophthalmic officer, Dip.; OO, male, statistician, MA) conducted the interviews. The two overarching questions were: (1) ‘*Now that you have been wearing your glasses for 6 months, can you tell me about your experience?’* and (2) ‘*What can you do now compared with before your vision was corrected?’*, with probes on economic, psychological, social and political improvement. The interviews were audio-recorded in a private room for 45–65 min per group and then manually transcribed verbatim, translated into English by the interviewers and back into Swahili (OO, statistician, male, MA; DM, monitoring and evaluation specialist, female, MSc).

Two analysts (CG, anthropologist, female, PhD; VFC, global health practitioner, male, PhD) conducted direct content analysis on the interviews. They independently coded transcripts according to the themes of economic, psychological, social and political improvement and provided a narrative to explain the quantitative results. The two analysts discussed the narratives when there were disagreements to reach a consensus. Due to the small cohort, there is a chance of jigsaw identification, where someone could be identified by combining various pieces of information. To protect anonymity, we reported qualitative illustrative quotes describing the interviewees’ profiles and abbreviated to focus group number; participant number (eg, focus group 1;female 1 is denoted as FG1;F1). The data underlying the results presented in the study are available from the Zenodo Data Repository.^26^

## Results

### Demographic profiles of craftswomen

**Figure 1:**
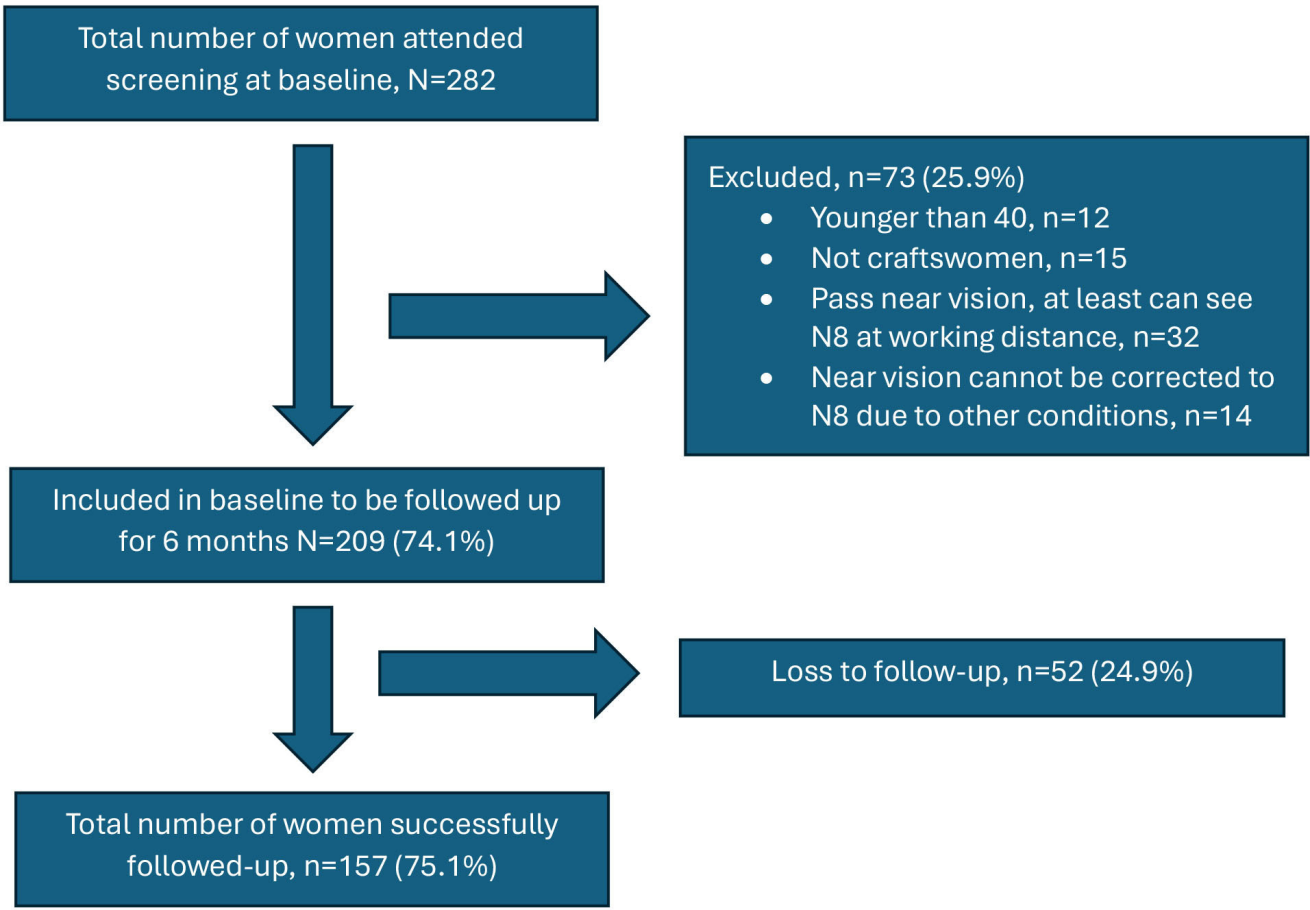
Description of the participant recruitment process from the initial screening and the final follow-up for analysis.

Even though 239 invitations were sent out to those 40 years old and older, 282 craftswomen attended the eye health screening. However, we excluded 73 who did not meet the inclusion criteria. Of those initially invited, 209 (87.4%) participated in the study. Another 49 (23.4%) were lost to follow-up due to non-response after three phone calls, and three reported their spectacles were stolen or broken. The final number of successfully followed-up and included in the analysis was 157 (75.1%) (figure 1). Demographic comparisons showed more craftswomen from Pemba than Unguja and five or more children among those successfully followed up than in the final analysis. Most of those followed-up were from Pemba Island (52.2%), 40–50 age group (50.2%), weavers (51.6%), worked 10 years or less in their craft (68.2%), married (64.5%) and supported 1–4 dependents (52.2%) (table 1).

**Table 1:** Craftswomen’s demographic characteristics of those successfully followed up and lost to follow-up

	Successfully followed upn (%)	Loss to follow-upn (%)	P value*
Locations			
Unguja	75 (47.8%)	36 (69.2%)	<0.005
Pemba	82 (52.2%)	16 (30.8%)	
Age group (years)			
40–50	79 (50.2%)	23 (44.2%)	0.471
51–60	63 (40.1%)	21 (40.4%)	
Older than 60	15 (9.6%)	8 (15.4%)	
Level of education			
No formal education	18 (11.6%)	6 (11.6%)	0.372
Did not complete primary education	20 (12.8%)	6 (11.6%)	
Completed primary education	66 (42.1%)	28 (53.9%)	
Completed secondary education	52 (33.2%)	11 (21.4%)	
Type of craftwork engaged			0.156
Weaving	81 (51.6%)	33 (63.5%)	
Tailoring and sewing	51 (32.5%)	15 (28.8%)	
Pottery	7 (4.50%)	3 (5.80%)	
Producing oil and making soaps	18 (11.5%)	1 (1.90%)	
Years working as a craftswoman			0.307
10 years and lesser	107 (68.2%)	33 (63.5%)	
>10–20 years	39 (24.8%)	15 (28.8%)	
>20–30 years	10 (6.40%)	2 (3.80%)	
>30–40 years	1 (0.60%)	2 (3.80%)	
Marital status			0.685
Single	2 (1.30%)	1 (1.90%)	
Married	117 (74.5%)	41 (78.8%)	
Widowed	20 (12.7%)	7 (13.5%)	
Separated/divorced	18 (11.5%)	3 (5.80%)	
Number of children			0.046
1–4	47 (30.4%)	12 (23.1%)	
5–8	74 (47.7%)	35 (67.3%)	
More than 8	32 (21.0%)	5 (9.60%)	
Number of dependents			0.366
None	8 (5.10%)	5 (9.60%)	
1–4	82 (52.2%)	31 (59.6%)	
5–8	61 (38.9%)	15 (28.8%)	
More than 8	6 (3.80%)	1 (1.90%)	
Prescription of near reading glasses			0.683
+1.00D to +1.75D	46 (29.3%)	12 (23.1%)	
+2.00D to +2.75D	74 (47.1%)	27 (51.9%)	
>+2.75D	37 (23.6%)	13 (25.0%)	
Total	157 (100%)	52 (100%)	

* Pearson Χ2chi-square test was used.

### Economic empowerment

Compared with the baseline, a higher proportion of craftswomen agreed or strongly agreed that they can run good businesses after being provided with near vision spectacles (OR 1.30; 95% CI 1.12 to 2.21, p<0.001). Furthermore, a significant increase in their ability to decide how they run their business was observed (OR 1.66; 95% CI 1.04 to 2.64, p<0.001).

Six months after correction, they felt they could earn enough income through their business (OR 1.30; 95% CI 1.06 to 2.26, p<0.001) and support their family financially (OR 1.20; 95% CI 1.09 to 2.22, p=0.003). Although the increase in their ability to improve their economic conditions after the provision of near vision spectacles was insignificant (OR 1.17; 95% CI 0.66 to 2.08, p=0.062), craftswomen felt that their financial situation was more stable (table 2).

**Table 2:** Self-reported women’s empowerment at baseline and follow-up (N=157) and OR of upward movement on the Likert scale

Statements		Strongly disagreen (%)	Disagreen (%)	Agreen (%)	Strongly agreen (%)	Odds ratio (95% CI)	P value
Economic empowerment							
I can run a good business	Baseline	6 (3.8%)	17 (10.8%)	108 (68.8%)	26 (16.6%)	1.30 (1.12 to 2.21)	<0.001
	Follow-up	2 (1.3%)	4 (2.5%)	95 (60.5%)	56 (35.7%)		
I can decide how I run my business	Baseline	2 (1.3%)	8 (5.1%)	101 (64.3%)	46 (29.3%)	1.66 (1.04 to 2.64)	<0.001
	Follow-up	1 (0.6%)	1 (0.6%)	65 (41.4%)	90 (57.3%)		
I can earn enough income through my business	Baseline	9 (5.7%)	28 (17.8%)	97 (61.8%)	23 (14.6%)	1.30 (1.06 to 2.26)	<0.001
	Follow-up	2 (1.3%)	2 (1.9%)	98 (62.4%)	54 (34.4%)		
I can support my family financially	Baseline	9 (5.7%)	28 (17.8%)	103 (65.6%)	17 (10.8%)	1.20 (1.09 to 2.22)	0.003
	Follow-up	3 (1.9%)	14 (8.9%)	100 (63.7%)	40 (25.5%)		
I can improve my economic conditions	Baseline	7 (4.5%)	19 (12.1%)	109 (69.4%)	22 (14.0%)	1.17 (0.66 to 2.08)	0.062
	Follow-up	2 (1.3%)	14 (8.9%)	99 (63.1%)	42 (26.8%)		
Social empowerment							
I am equal to my peers	Baseline	3 (1.9%)	26 (16.6%)	83 (52.9%)	45 (28.7%)	2.24 (1.38 to 3.63)	<0.001
	Follow-up	2 (1.3%)	10 (6.4%)	38 (24.2%)	107 (68.2%)		
I am brave enough to voice my opinion in public	Baseline	2 (1.3%)	6 (3.8%)	90 (57.3%)	59 (37.6%)	1.72 (1.09 to 2.72)	<0.001
	Follow-up	0 (0.0%)	3 (1.9%)	54 (34.4%)	100 (63.7%)		
I can make decisions for my children	Baseline	2 (1.3%)	9 (5.7%)	80 (51.0%)	66 (42.0%)	1.57 (1.00 to 2.47)	0.003
	Follow-up	1 (0.6%)	4 (2.5%)	53 (33.8%)	99 (63.1%)		
I can make decisions for my household/family	Baseline	2 (1.3%)	6 (3.8%)	86 (54.8%)	63 (40.1%)	1.29 (0.82 to 2.02)	0.097
	Follow-up	2 (1.3%)	2 (1.3%)	69 (43.9%)	84 (53.5%)		
Psychological empowerment							
I am at peace with myself	Baseline	3 (1.9%)	17 (10.8%)	71 (45.2%)	66 (42.0%)	2.68 (1.63 to 4.39)	<0.001
	Follow-up	1 (0.6%)	0 (0.00%)	33 (21.0%)	123 (78.3%)		
I feel that I am a person of worth	Baseline	1 (0.6%)	2 (1.3%)	73 (46.5%)	81 (51.6%)	1.73 (1.08 to 2.76)	0.004
	Follow-up	0 (0.0%)	3 (1.9%)	41 (26.1%)	113 (72.0%)		
I feel I have much to be proud of	Baseline	3 (1.9%)	3 (1.9%)	79 (50.3%)	72 (45.9%)	1.15 (0.74 to 1.79)	0.155
	Follow-up	1 (0.6%)	10 (6.4%)	63 (40.1%)	83 (52.9%)		
I understand my capabilities	Baseline	0 (0.0%)	5 (3.2%)	85 (54.1%)	67 (42.7%)	1.36 (0.87 to 2.13)	0.093
	Follow-up	0 (0.0 %)	4 (2.5%)	62 (39.5%)	91 (58.0%)		
I understand my need	Baseline	0 (0.0%)	4 (2.5%)	76 (48.4%)	77 (49.0%)	1.63 (1.03 to 2.58)	0.004
	Follow-up	0 (0.0%)	0 (0.0%)	49 (31.2%)	108 (68.8%)		
Political empowerment							
I can advise on different situations in my community	Baseline	3 (1.9%)	10 (6.4%)	85 (54.1%)	59 (37.6%)	2.24 (1.43 to 3.51)	0.016
	Follow-up	0 (0.0%)	2 (1.3%)	74 (47.1%)	81 (51.6%)		
I can be elected as a leader	Baseline	20 (12.7%)	23 (14.6%)	65 (41.4%)	49 (31.2%)	1.57 (0.93 to 2.65)	0.316
	Follow-up	15 (9.6%)	32 (20.4%)	48 (30.6%)	62 (39.5%)		
I can elect someone capable of becoming a leader	Baseline	1 (0.6%)	4 (2.5%)	80 (51.0%)	72 (45.9%)	1.72 (1.08 to 2.74)	<0.001
	Follow-up	0 (0.0%)	1 (0.60%)	25 (15.9%)	131 (83.4%)		
I can advise government leaders	Baseline	4 (2.5%)	9 (5.7%)	87 (55.4%)	57 (36.3%)	1.29 (0.82 to 2.03)	0.07
	Follow-up	5 (3.2%)	21 (13.4%)	59 (37.6%)	72 (45.9%)		

Improved vision transforms both business practices and personal lives by providing individuals with the autonomy to manage their economic activities and improve their financial stability. As described, the ability to ‘*do our business the way we want’ (FG1;F2*) allows for effective control over production and financial decisions, enabling individuals to ‘*earn money that we can satisfy and pay the costs of the products and provide the equipment to make the product’ (FG1;F2*). This newfound control leads to tangible improvements.

Our work has improved, and we have grown very strong in production… The profits have improved, which leads to an increase in income. (FG3;F15)

The impact of this economic progress extends beyond business success. Increased income bolsters self-confidence and facilitates family support. One participant noted,

I have self-confidence after the income increases and can support my family (FG2;F8)

while another highlights the personal fulfilment of contributing to household expenses and buying items for children:

I can contribute to the household, and also buy the children even small things and run the family well. (FG3;F16)

Collectively, these advancements culminate in greater economic stability, as reflected in the sentiment,

so I have more income and becoming more economically stable. (FG1;F5)

### Social empowerment

Six-month post-presbyopia correction, significantly more craftswomen felt they were equal to their peers (OR 2.24; 95% CI 1.38 to 3.63, p<0.001) and brave enough to voice their opinion in public (OR 1.72; 95% CI 1.09 to 2.72; p<0.001). Significantly more craftswomen felt that they could decide for their children (OR 1.57; 95% CI 1.00 to 2.47; p=0.003), while the increase in ability to decide for the family was insignificant (p=0.097) (table 2).

In the past 6 months, improved vision was seen to have led to social empowerment in terms of personal and communal transformations. Individuals have gained confidence and the ability to contribute meaningfully to their families and communities. One participant notes, ‘*For me, (the glasses) have helped me a lot in 6 months and … I find time to understand people’s situations and give my advice to them’ (FG2;F8),* highlighting how personal growth fosters support and engagement with others. This empowerment starts with increased self-confidence, achieved by reducing reliance on external factors and taking control of one’s decisions. As one person reflects, ‘*My confidence has increased since my vision got better. I have reduced dependence on the number of issues… It is enough for me to be a leader of my children’ (FG2;F6),* showing a shift towards personal leadership and broader social change.

With growing confidence, the understanding of shared family responsibilities has evolved. Traditionally, one member, often the husband, made decisions, but now, ‘*It is a shared responsibility to decide for the family now’ (FG2;F9*). This change promotes equitable power distribution and mutual respect.

### Psychological empowerment

In terms of psychological empowerment, significantly more craftswomen were at peace with themselves (OR 2.68; 95% CI 1.63 to 4.39, p<0.001), felt worthy (OR 1.73; 95% CI 1.08 to 2.76, p=0.004) and understood their needs (OR 1.63; 95% CI 1.03 to 2.58, p=0.004). Although increases were observed in the number of craftswomen feeling proud of themselves (p=0.155) and understanding their capabilities (p=0.093), these increases were insignificant (table 2).

As individuals encounter more opportunities with better vision, their self-assurance grows, enabling them to engage more actively in decision-making. This newfound confidence transforms from a vague feeling into a powerful force, driving them to seek and embrace new chances. One participant noted:

Before I got my glasses, I always relied on others to help me with my work, but now I can do it myself. I feel more confident, and when decisions are being made about our group, I speak up more because I know I can contribute just as much as anyone else. (FG1;F3)

This illustrates the shift from passive waiting to proactive anticipation, marking a significant change in how people perceive their ability to shape their futures. Another participant emphasised the practical impact of this empowerment:

I used to wait for someone to tell me about opportunities or help me with my business, but now I’m looking for new ways to grow. I’ve started asking questions, looking for markets, and thinking about how I can expand on my own. (FG2;F8)

### Political empowerment

Regarding political empowerment, there was a significant increase in those who felt they could advise their community on different situations (OR 2.24; 95% CI 1.43 to 3.51, p=0.016) after they were corrected. Significantly more craftswomen felt they could elect other capable candidates to leadership roles (OR 1.72; 95% CI 1.08 to 2.74, p<0.001). In contrast, significantly more craftswomen did not feel that having better vision improved their ability to be elected as leaders (p=0.316) or that they could give advice to the government (p=0.07), even though interviews showed two opposing themes of them having the confidence to participate in leadership roles and those who were not interested in leadership roles (table 2).

The effects of better vision on political empowerment are exemplified by the ability to actively engage in community decisions and assume leadership roles, reflecting individual confidence and a commitment to collective well-being. As one participant noted:

… since I can see well, and if a problem occurs, I can help to solve the problems with my community leaders, other women, and even men! I can participate in meetings and unexpected situations as well. (FG2;F6)

This illustrates how improved visibility and involvement in community issues foster a sense of responsibility and capability. Participants also express a strong sense of confidence in their roles within the community, stating:

I can see better. I can work better… we are very confident in making contributions to the community and making various decisions. (FG1;F1)

This confidence is further enhanced by their ability to engage in decision-making processes, such as choosing representatives for community roles:

I can participate in choosing someone to participate in various things in the community. (FG3;F14)

Political empowerment also includes the capacity to make personal decisions and assume leadership positions. One participant highlights:

… for me, it also helps me a lot to decide my affairs, and I can participate in the leadership. (FG2;F10)

They also expressed readiness to face public responsibilities, saying

With the reading glasses, I can read and not [seen as] uneducated. I can appear in public without any problem and be able to decide anything, and also, if I am elected to be a leader, I have confidence that I will be able to perform better. (FG2;F6)

However, political empowerment is not universally sought after. As noted,

… not interested to lead. I am happy with my current situation… (FG3;F17)

[leadership] is not for everyone. (FG2;F10)

This underscores the diverse perspectives on leadership and participation within the community, acknowledging that while some embrace these roles with enthusiasm, others are content with their current engagement levels.

## Discussion

The present study aimed to evaluate the effect of presbyopia correction on older Zanzibari craftswomen’s self-reported and perceived empowerment following 6 months of spectacle wear across four domains: economic, psychological, social and political. The findings show that craftswomen experience greater empowerment across all domains after correcting their presbyopia.

Our findings provide further evidence that spectacle correction can improve the confidence and autonomy of craftswomen in how they run their businesses, which is economically important in LMICs. Existing evidence^6^,^911 13^ from LMICs suggests that uncorrected presbyopia affects many economically important activities and that correction could lead to improvement in productivity. It is also imperative for craftswomen to have an optimistic outlook on their businesses, as this is positively linked to business growth.^27 28^ Improving their financial situation by being able to make decisions on spending, saving and investing in their business is also highly relevant to their lives.

Furthermore, the findings on the increased self-confidence among the craftswomen in expressing their opinions and feeling equal to their peers are paramount in helping Zanzibari women overcome obstacles in accessing financial assistance. These obstacles include fear of inquiring about bank loans, lack of confidence in negotiating loan conditions, the Islamic prohibition on loans and interest payments, and discrimination from bank officials.^21 29^ Instilling confidence in these women can significantly aid in overcoming the challenges they face in their business endeavours.

Acknowledging that local social and cultural factors may significantly impact women’s perceptions of empowerment is imperative. Research suggests that some women may hesitate to pursue certain tasks due to a lack of confidence in their abilities.^30^ However, our study demonstrated that presbyopia correction can enhance the craftswomen’s self-worth, self-peace and understanding of needs. As education becomes more widely available, traditional gender roles may shift, leading to changes in attitudes toward gender differences and equality.^31^ Improving the self-esteem and self-efficacy of our study cohort is of utmost importance, as it may help address the deeply ingrained self-limiting behaviours that women often experience.

Craftswomen reported minimal improvement regarding political empowerment, as it requires significant consideration and support from the wider community, which can take many years to achieve. Additionally, some craftswomen stated that not everyone is suited to be a leader. However, one long-term goal is to enhance self-organisation and self-efficacy among craftswomen, which could increase their visibility, recognition and voice^32 33^ in an unfavourable environment for women entrepreneurs. Cultural factors may have hindered the achievement of political empowerment, and leadership skills and confidence require a longer period to develop. Therefore, the short follow-up period of the current study may not fully capture the impact of empowerment among craftswomen.

While the ORs indicate statistically significant changes in empowerment, it is important to consider the practical implications of these findings in the participants’ everyday lives. The accompanying qualitative data provide valuable context, showing that vision correction led to tangible improvements in the craftswomen’s productivity, income generation and self-confidence. Participants reported feeling more empowered to engage in their crafts and business activities, suggesting that the changes in empowerment are not merely statistical, but are experienced as meaningful improvements in their social and economic well-being.

Our study has limitations that must be acknowledged. Our study sample’s unique characteristics may limit our results’ generalisability to other work settings. Additionally, ethical considerations prevented us from conducting a trial by withholding an effective and safe intervention from the women. Without a control group, our study’s quantitative results may be influenced by confounding factors such as changes in social engagement, economic opportunities or household dynamics unrelated to the intervention. Furthermore, using a self-reported and positive phrasing questionnaire in this study may have introduced respondent bias and overestimated the intervention effects. However, our mixed-method approach—combining quantitative data and qualitative insights—helps provide a holistic view of the empowerment process. A short follow-up may not fully reflect the intervention’s effects, as empowerment involves gradual changes in social roles, economic opportunities and self-perception. Overcoming cultural norms and stereotypes can take time.^34 35^ Measuring empowerment requires assessing self-esteem, decision-making, economic independence and social status, which may take longer than 6 months to change.

## Conclusion

The correlation between a simple spectacle correction and empowerment is a complex one.^23^ Simply providing spectacle correction alone will not address all gender-related issues among the craftswomen, as cultural and social factors also play a role. However, the findings from this study demonstrate that providing eye care services can positively impact the economic stability of craftswomen and their families, as well as their social and psychological well-being.

## supplementary material

10.1136/bmjopen-2024-086624 online supplemental file 1

10.1136/bmjopen-2024-086624 online supplemental file 2

10.1136/bmjopen-2024-086624 online supplemental file 3

## Data Availability

Data are available in a public, open access repository.

## References

[1] Bourne RRA, Flaxman SR, Braithwaite T (2017). Magnitude, temporal trends, and projections of the global prevalence of blindness and distance and near vision impairment: a systematic review and meta-analysis. Lancet Glob Health.

[2] Fricke TR, Tahhan N, Resnikoff S (2018). Global Prevalence of Presbyopia and Vision Impairment from Uncorrected Presbyopia: Systematic Review, Meta-analysis, and Modelling. Ophthalmology.

[3] Holden BA, Fricke TR, Ho SM (2008). Global vision impairment due to uncorrected presbyopia. Arch Ophthalmol.

[4] He M, Abdou A, Ellwein LB (2014). Age-related prevalence and met need for correctable and uncorrectable near vision impairment in a multi-country study. Ophthalmology.

[5] Goertz AD, Stewart WC, Burns WR (2014). Review of the impact of presbyopia on quality of life in the developing and developed world. Acta Ophthalmol.

[6] Lu Q, Congdon N, He X (2011). Quality of Life and Near Vision Impairment Due to Functional Presbyopia among Rural Chinese Adults. Invest Ophthalmol Vis Sci.

[7] Patel I, Munoz B, Burke AG (2006). Impact of presbyopia on quality of life in a rural African setting. Ophthalmology.

[8] Nirmalan PK, Krishnaiah S, Shamanna BR (2006). A Population-Based Assessment of Presbyopia in the State of Andhra Pradesh, South India: The Andhra Pradesh Eye Disease Study. Invest Ophthalmol Vis Sci.

[9] World Economic Forum (2016). Eyeglasses for global development: bridging the visual divide.

[10] Ahmed M, Shefali MK, Husain L (2022). Vision Impairment and Productivity Among Female Garment Workers in Bangladesh: A Cohort Study. Asia Pac J Ophthalmol (Phila).

[11] Frick KD, Joy SM, Wilson DA (2015). The Global Burden of Potential Productivity Loss from Uncorrected Presbyopia. Ophthalmology.

[12] Reddy PA, Congdon N, MacKenzie G (2018). Effect of providing near glasses on productivity among rural Indian tea workers with presbyopia (PROSPER): a randomised trial. Lancet Glob Health.

[13] Naidoo KS, Jaggernath J, Chinanayi FS (2016). Near vision correction and work productivity among textile workers. Afr Vis Eye Health.

[14] Pradhan KB, Professor, Chitkara school of Health sciences, Chitkara university, Punjab (2015). Impact of Uncorrected Vision on Productivity-A study in an Industrial setting a Pair of Spectacles. *JMRH*.

[15] Dalberg Global Development Advisors (2015). Impact study of Essilor’s Eye Mitra optician programme in India (a programme of the Essilor Group’s 2.5 New Vision Generation division).

[16] Laviers HR, Omar F, Jecha H (2010). Presbyopic spectacle coverage, willingness to pay for near correction, and the impact of correcting uncorrected presbyopia in adults in Zanzibar, East Africa. Invest Ophthalmol Vis Sci.

[17] Chan VF, Naidoo J, Chinanayi FS (2017). Near vision correction and quality of life among textile factory workers in Durban. Afr Vis Eye Health.

[18] Chan VF, MacKenzie GE, Kassalow J (2019). Impact of Presbyopia and Its Correction in Low- and Middle-Income Countries. *Asia Pac J Ophthalmol (Phila*).

[19] Burton MJ, Ramke J, Marques AP (2021). The Lancet Global Health Commission on Global Eye Health: vision beyond 2020. Lancet Glob Health.

[20] Bourne R, Steinmetz JD, Flaxman S (2021). Trends in prevalence of blindness and distance and near vision impairment over 30 years: an analysis for the Global Burden of Disease Study. Lancet Glob Health.

[21] UN Women Data Hub (2019). Women and men in Zanzibar: Facts and Figures. https://data.unwomen.org/publications/women-and-men-zanzibar-facts-and-figures.

[22] Chan VF, Omar F, Farmer A (2023). Refractive error, eye care needs and attitude towards spectacle wearing among older Zanzibari craftswomen and implications for developing women-targeted services: a cross-sectional study. *BMJ Open Ophth*.

[23] Martins MF, Omar F, Othman O (2023). How does a pair of near-vision spectacle correction empower older Zanzibari craftswomen?: A qualitative study on perception. PLoS One.

[24] Sebayang SK, Ferry E, Erni A (2017). Women’s empowerment and the use of antenatal care services in Southeast Asian countries.

[25] Sen G (1997). Empowerment as an approach to poverty, working paper series 97.07, background paper for the UNDP human development report.

[26] Chan VF, Omar F, Othman O Women’s Empowerment through Investing in Zanzibari Craftswomen’s Eyesight (Version 1.0.0).

[27] De Jongh J, Mncayi P (2018). An econometric analysis on the impact of busines confidence and investment on economic growth in post-apartheid South Africa. Int J Econ Financ Stud.

[28] Santero T, Westerlund N (1996). Confidence indicators and their relationship to changes in economic activity.

[29] Ellis A (2007). Gender and economic growth in Tanzania: Creating opportunities for women.

[30] Dickerson A, Taylor MA (2000). Self-Limiting Behavior in Women. Grp & Org Mgmt.

[31] Stewart R, Wright B, Smith L (2021). Gendered stereotypes and norms: A systematic review of interventions designed to shift attitudes and behaviour. Heliyon.

[32] Quak E, Barenboim I (2002). Female Entrepreneurship and Informality in Low- and Middle-Income Countries: What Have We Learned So Far.

[33] Krapfl JE, Blina K (2018). Leadership and culture. Leadership and cultural change.

[34] Kabeer N, Mahmud S, Tasneem S (2018). The Contested Relationship Between Paid Work and Women’s Empowerment: Empirical Analysis from Bangladesh. *Eur J Dev Res*.

[35] Kabeer N (1999). Resources, Agency, Achievements: Reflections on the Measurement of Women’s Empowerment. Dev Change.

