# Supplementary material for: Correcting near vision impairment and women’s empowerment: a before-after mixed-methods study among older Zanzibari craftswomen
Source: BMJ Open. 2024 Nov 14;14(11):e086624. doi: 10.1136/bmjopen-2024-086624 (PMC11575388; doi:10.1136/bmjopen-2024-086624)
Supplement: online supplemental file 1 [file bmjopen-14-11-s001.pdf]

Supplemental material 1: Average relevance score, factor analysis and the inclusion and exclusion of empowerment statements

Economic	Average relevance score	Factor analysis	Included in survey (Yes/No)
I can run a good business.	3.7	0.856	Yes
I can decide how I run my business	3.5	0.893	Yes
I can earn enough income through my business.	3.8	0.974	Yes
I can support my family financially.	4.3	0.878	Yes
I can improve my economic conditions.	4.7	0.901	Yes
**Social**			
I believe in my abilities.	2.2	0.678	No
I can make decision for myself.	2.9	0.496	No
I can make decision for my children.	3.6	0.712	Yes
I can make decision for my household/family.	3.8	0.811	Yes
I am equal to my peers (e.g. sister, friends, colleagues, etc.)	3.3	0.776	Yes
I can identify victims of harassment	1.9	[redacted]	No
I can co-parent my child/children.	3.2	0.324	No
I am brave enough to voice my opinion.	3.3	0.898	Yes
I can protect myself from sexual harassment.	2.9	0.628	No
**Psychological**			
I am at peace with myself.	4.1	0.898	Yes
I understand myself.	1.8	[redacted]	No
I feel that I am a person of worth	3.2	0.726	Yes
I feel that I have many good qualities	2.1	0.568	No
I feel I have much to be proud of	3.9	0.742	Yes
I understand my capabilities.	4.3	0.910	Yes
I understand my needs.	2.7	0.811	Yes
**Education**			
I can access the business/entrepreneur market.	2.8	0.682	No
I can be think creatively producing different products	1.8	[redacted]	No
I can give support to other craftswomen	1.9	[redacted]	No
I can visit my fellow entrepreneurs to exchange ideas.	3.1	0.678	No
**Political**			
I can give advice on different situations in my community.	3.6	0.816	Yes
I can be elected as a leader.	3.7	0.709	Yes

I can elect someone capable to become a leader.	3.4	0.718	Yes
I can advise to government leaders.	2.6	0.701	Yes
I can follow governments guidelines on entrepreneurship.	1.1	[redacted]	No
