# Supplementary material for: Correcting near vision impairment and women’s empowerment: a before-after mixed-methods study among older Zanzibari craftswomen
Source: BMJ Open. 2024 Nov 14;14(11):e086624. doi: 10.1136/bmjopen-2024-086624 (PMC11575388; doi:10.1136/bmjopen-2024-086624)
Supplement: online supplemental file 2 [file bmjopen-14-11-s002.pdf]

Supplemental material 2: Survey form

Demography **Unique study** |__|__|__|__|__|__|__|__|

Section 1: Preamble

1.1. Interviewer Name: ________________

1.2. Date: |__|__|__|__|YYYY/|__|__|MM/|__|__|DD

1.3 Presenting distance vision at 6/12: RE: Pass/Fail LE: Pass/Fail

1.4 Prescription distance: RE____/_____X____ LE_____/_____x____

1.5 Corrected vision (to be captured from patient record): R________ L_________

1.6 Presenting near vision at N8 at 40cm: RE: Pass/Fail LE: Pass/Fail

1.7 Presenting near vision at N8 at working distance: RE: Pass/Fail LE: Pass/Fail

1.8 Final near prescription: RE ___________ LE ____________

1.9 Eye health examination: RE: Pass/Fail LE: Pass/Fail

1.10 If fail, what is the reason? ____

DO NOT READ: *Please fill out the preamble and read the instructions in the preamble BEFORE speaking to a participant. The whole preamble is not to be read out.*

Hello, my name is _______. I work as part of a research team interested in learning about your wellbeing and vision corrections. I would like to ask you some questions about vision health topics.

1.7. Respondent Name: _______________

Study participation consent statement here.

DO NOT READ: proceed to provide a written consent script

1.8. Are you interested in taking up this survey?

a. No.--> *Please end the survey and move on to the next participant.*
b. Yes.

Section 2: Screening

2.1. Was the respondent diagnosed with presbyopia?

a. Yes
b. No

At this point, we will know if they satisfy the inclusion criteria. If they do, the surveyor moves on to the next section.

2.2. Do you currently own a pair of glasses?

Note: "Own" means having a pair of usable glasses in their possession. The glasses do not have to be with them at work. Even if they have a pair of glasses in a usable condition at home, they own the glasses.

a. Yes
b. No

998. Other, specify:_____.

Section 3: Demographics and Contact Details

3.1. Are you married or unmarried?

a. Unmarried
b. Married
c. Widowed
d. Separated/Divorced

998. Other (specify) ___________________________

3.2. Do you have any children?

a. Yes
b. No

3.3. {If 3.2.= a}How many children do you have? ____________

3.4. How many people live in your household and rely on your income? ______

3.5. Do you have access to a mobile phone?

a. Yes
b. No

3.6. {If 3.5.=a} What is your mobile phone number? ________

To what extent do you agree with the following statement?	Strongly disagree (1)	Partly disagree (2)	Partly agree (3)	Strongly agree (4)
**Economic**				
I can run a good business.				
I can decide how I run my business				
I can earn enough income through my business.				
I can support my family financially.				
I can improve my economic conditions.				
**Social**				
I can make decision for my children.				
I can make decision for my household/family.				
I am equal to my peers (e.g. sister, friends, colleagues, etc.)				
I am brave enough to voice my opinion.				
**Psychological**				
I am at peace with myself.				
I feel that I am a person of worth				
I feel I have much to be proud of				
I understand my capabilities.				
I understand my needs.				
**Political**				
I can give advice on different situations in my community.				
I can be elected as a leader.				
I can elect someone capable to become a leader.				
I can advise to government leaders.				
