# Supplementary material for: Correcting near vision impairment and women’s empowerment: a before-after mixed-methods study among older Zanzibari craftswomen
Source: BMJ Open. 2024 Nov 14;14(11):e086624. doi: 10.1136/bmjopen-2024-086624 (PMC11575388; doi:10.1136/bmjopen-2024-086624)
Supplement: online supplemental file 3 [file bmjopen-14-11-s003.pdf]

Supplemental material 3: Interview Guide for craftswomen who were provided with presbyopia correction

ICEBREAKER - PURPOSE: PUT PARTICIPANTS AT EASE BEFORE ASKING KEY QUESTIONS

1. Good. Let us get started. I would like again to welcome everyone here. You may know each other, but I do not know any of you. So would each of you briefly introduce yourself? Tell us your first name, and what do you do?

CORE QUESTION #1 – PURPOSE: IDENTIFY HOW PRESBYOPIA CORRECTION AFFECT THEM

1. **Now that you have been wearing your glasses for 6 months, can you tell me about your experience?**

CORE QUESTION # 2 – PURPOSE: IDENTIFY HOW PRESBYOPIA CORRECTION EMPOWER THEM

2. How about good experiences? What can you do NOW compared to before your vision was corrected?
    - PROBES: More income? Savings? Buy things that they wanted to buy for their kids, themselves, or family? Having the power to decide on how to spend money? Contributes more to household income?
    - Self-esteem? Self-confident? More time spent with friends and family/socializing?
    - Involve in deciding for the household?
    - Participation in public leadership? Participation in public events or community groups?
    - Reduced time in care responsibilities?

WRAP UP – PURPOSE: FINAL THOUGHTS & DEMOGRAPHIC/BACKGROUND INFORMATION

1. We are almost at the end of our meeting today. Thinking back to everything we talked about, is there anything you think I left out or did not ask you that would be important to know?
2. Is there anything you would like to talk about in more detail?

Thank you all again for taking part in the discussion today. Your contributions have all been helpful. Just to remind you, if there is anything you do not wish to be transcribed, please do have a word with me before you leave.
