# Supplementary material for: Correcting near vision impairment and women’s empowerment: a before-after mixed-methods study among older Zanzibari craftswomen
Source: BMJ Open. 2024 Nov 14;14(11):e086624. doi: 10.1136/bmjopen-2024-086624 (PMC11575388; doi:10.1136/bmjopen-2024-086624)
Supplement: Supplementary file 4 [file bmjopen-2024-086624.reviewer_comments.pdf]

PEER REVIEW HISTORY

BMJ Open publishes all reviews undertaken for accepted manuscripts. Reviewers are asked to complete a checklist review form and are provided with free text boxes to elaborate on their assessment. These free text comments are reproduced below.

ARTICLE DETAILS

Title (Provisional)

Correcting near vision impairment and women's empowerment: a before-after mixed-methods study among older Zanzibari craftswomen

Authors

Omar, Fatma; Othman, Omar Juma; Yong, Ai Chee; McConnell, Emma; Mulewa, Damaris; Graham, Christine; Shah, Kajal; Martins, Michelle Fernandes; Farmer, Adrianna; Graham, Ronnie; Mashayo, Eden; Chan, Ving Fai

---

VERSION 1 - REVIEW

**Reviewer**	**1**
**Name**	**Ramani, Krishna**
**Affiliation**	**Elite School of Optometry, Optometry**
**Date**	**21-May-2024**
**COI**	**NIL**

I like to congratulate all the authors for looking into the impact of presbyopia correction on women's empowerment , in all possible dimensions possible. I also felt the challenge in handling the mixed method designs. I also felt impressed with the flow of thought throughout the manuscript. Though i could appreciate the adequacy of information and data in quantitative study, I felt the same was not seen in qualitative study information. So I like to suggest you to look into possibility of split the content into 2 manuscripts and resubmit as part 1 and part 2

Comments

This manuscript on Impact of near vision correction on women's empowerment, beyond increased productivity and improved vision related quality of life, was interesting study outcome. I totally agree with the justification for such study among the population to ensure equality, aligning with Sustainable Development growth. However, I have my reservation or objection in adopting mixed method design. I will strongly recommend to resubmit as 2 manuscripts with part 1 quantitative study and part 2 Qualitative study.

1. Abstract:
    1.1. Line 30: Author can remove the word Noncontrolled.

1.2. Line 30-37: Rewrite the sentences. This non-controlled, mixed-method, before-after, intervention study included craftswomen 40 years and older with correctable presbyopia. First, they answered a questionnaire on economic, social, psychological and political empowerment, were corrected with glasses and followed up for six months, at which point the empowerment questionnaire was readministered. The four-point Likert scale responses were compared pre- and post35 correction. Subsequently, five focus group interviews were conducted to explore the craftswomen's daily experiences concerning vision correction and empowerment. Interviews were transcribed, translated and analysed using direct content analysis.
Suggestion: This mixed-method intervention study recruited Zanzibari craftswomen of 40 years of age and more, and provided with presbyopic correction following eye examination. Questionnaire on empowerment was administered before dispensing near vision spectacle and after 6 months for all those recruited in the study. Focus group discussions among craftswomen were conducted to explore the experiences of vision correction and empowerment. The discussions were transcribed, translated and analysed using direct content analysis.
1.3. Line 39,40: Rewrite the sentence with the most appropriate information. Craftswomen reported significantly greater economic, social, psychological and political empowerment in 14/18 statements (77.8%) ($p<0.05$)
Suggestion: Since you have mention about Likert scale, it is naturally expected to give the total score out of maximum marks before and after presbyopic correction along with the p value. In the abstract it is fine to give total scores.
1.4. Conclusion: Wonder whether the direct relation between presbyopic correction and empowerment was not influenced by any other confounders at all? It might be parallel occurrence too. How author managed to control the confounders that would have impacted the outcome.

2. Introduction: No changes required.
3. Methods: Rationale for why mixed method was adopted to answer the research question can be added. However, my suggestion to the author is to split this into two manuscripts. For this manuscript the authors can handle only the quantitative data. The challenge in combined study design is clearly seen in the manuscript through lean representation of the qualitative component or information.
4. Results and discussion: I have no comments for the quantitative data and its aligned discussion points. I would recommend the authors to rewrite the whole manuscript removing the qualitative aspect and make changes accordingly.

**Reviewer**	**2**
**Name**	**Dickinson, Chris**
**Affiliation**	**Manchester University**
**Date**	**08-Jul-2024**
**COI**	**None**

This is an interesting study which appears to follow on from earlier work by the same research group on empowerment and near vision correction (references 22 and 23).

The Introduction clearly sets out the aim of the study – to test the hypothesis of improvement in self-reported empowerment using a questionnaire, and to use focus groups to further understand and interpret these findings. Some of the sections in the Introduction are a bit cryptic, and it isn't clear what point the authors are trying to make: in particular lines 79-83 and lines 88-90.

It was surprising that greater use did not seem to be made of the findings of reference 23 in developing the questionnaire. Nonetheless the methodology appears reasonable, although the questionnaire was not validated. It is also not an ideal design that all questions are phrased in the same positive way, rather than having some where the "positive" response would have been to disagree with the statement.

The process of provision of spectacles is not very clear. Did any of these women have uncorrected distance refractive errors and were these corrected as well? Was any payment required from the women for the spectacles (and is there a sustainable plan to continue to supply in the future?).

What was the approximate response rate to the initial invitation to the eye health screening – how many women were approached to participate? Were the questionnaires conducted verbally or in writing?

How were the participants for the focus group selected? The questions asked in the focus group don't appear to relate to empowerment. The sessions are then referred to as interviews? The 30 minute duration seems very short for a focus group with facilitated discussion. Did these take place after the follow-up questionnaire? The presentation of the results of this part of the study is very disappointing. The method describes coding and developing themes, but these are not really apparent in the presentation – there just seems to be a selection of a few quotes, some of which are not particularly illustrative of the point being made (eg lines 221-222, and lines 232-234), and I don't believe they fulfil the aim of explaining the questionnaire responses. The "key messages" statement that "craftswomen also provided detailed accounts of how their lives have transformed" also rather overstates what is reported in the paper.

In the results on Line 187 (and in the abstract) shouldn't the 157 participants be 75.1% rather than 74.1%?

In the quantitative results, it is confusing that the text does not use the same wording as the questions. There are numerous examples of this: eg in the text “agreed that they were able to effectively manage their businesses and make important decisions” but the question was actually “I can run a good business”.

The Discussion does not address any issues with confounders. For example, what is meant in lines 137-140 by “promoting empowerment”? Could the women have been influenced by factors other than the spectacles? Would they have wanted to be positive about the effect of spectacles to please the researchers? It would also be worth discussing that many aspects of empowerment also appeared high before the intervention – typically 80-90% of the responses were agree or strongly agree, even at baseline. Therefore, even if the results are statistically significant, are they actually practically significant in changing levels of empowerment? The limitations of the questionnaire design should also be addressed.

The authors should take care not to go too far into speculation in their Discussion (and in the conclusion of the Abstract). Its not clear what evidence they have for needing a longer timeframe to assess results. They should also be careful in their comments hinting at entrepreneurship and mentoring, since three questions on this issue (and a couple more in the “education” category which are related) were removed from the final questionnaire.

VERSION 1 - AUTHOR RESPONSE

Reviewer: 1 [PLEASE SEE ATTACHED FILE FOR ADDITIONAL COMMENTS FROM REVIEWER 1]

Dr. Krishna Ramani, Elite School of Optometry

Comments to the Author:

I like to congratulate all the authors for looking into the impact of presbyopia correction on women's empowerment , in all possible dimensions possible. I also felt the challenge in handling the mixed method designs. I also felt impressed with the flow of thought throughout the manuscript. Though i could appreciate the adequacy of information and data in quantitative study, I felt the same was not seen in qualitative study information. So I like to suggest you to look into possibility of split the content into 2 manuscripts and resubmit as part 1 and part 2.

Response: Thank you for the feedback. Please see detailed responses below.

Abstract:

1.1. Line 30: Author can remove the word Noncontrolled.

Response: Amended.

1.2. Line 30-37: Rewrite the sentences. This non-controlled, mixed-method, beforeafter, intervention study included craftswomen 40 years and older with

correctable presbyopia. First, they answered a questionnaire on economic, social, psychological and political empowerment, were corrected with glasses and followed up for six months, at which point the empowerment questionnaire was readministered. The four-point Likert scale responses were compared pre- and post35 correction. Subsequently, five focus group interviews were conducted to explore the craftswomen's daily experiences concerning vision correction and empowerment. Interviews were transcribed, translated and analysed using direct content analysis. Suggestion: This mixed-method intervention study recruited Zanzibari craftswomen of 40 years of age and more, and provided with presbyopic correction following eye examination. Questionnaire on empowerment was administered before dispensing near vision spectacle and after 6 months for all those recruited in the study. Focus group discussions among craftswomen were conducted to explore the experiences of vision correction and empowerment. The discussions were transcribed, translated and analysed using direct content analysis.

Response: We have reformatted the abstract according to the journal format and this is now omitted to avoid repetition of the methods.

1.3. Line 39,40: Rewrite the sentence with the most appropriate information. Craftswomen reported significantly greater economic, social, psychological and political empowerment in 14/18 statements (77.8%) (p<0.05) Suggestion: Since you have mention about Likert scale, it is naturally expected to give the total score out of maximum marks before and after presbyopic correction along with the p value. In the abstract it is fine to give total scores.

Response: We politely disagree with this suggestion for the following reasons:

a. Likert scale data is ordinal, meaning the order of the responses matters, but the differences between them are not equal. Converting to a numerical score implies equal intervals, which is inaccurate
b. Calculating total scores from Likert data can be misleading as it implies equal intervals between response options.
c. The p-value quoted was obtained based on the fact that these are ordinal data and hence non-parametric test designed for ordinal data to understand the correlation.

1.4. Conclusion: Wonder whether the direct relation between presbyopic correction and empowerment was not influenced by any other confounders at all? It might be parallel occurrence too. How author managed to control the confounders that would have impacted the outcome.

Response: We acknowledge that, in the absence of a control group, our study's quantitative results may be influenced by confounding factors such as changes in social engagement, economic opportunities, or household dynamics unrelated to the intervention. However, we believe our mixed-method approach—combining both quantitative data and qualitative insights—helps provide a holistic view of the empowerment process (clarified now in LINE). The qualitative findings indicate that participants explicitly attributed improvements in their economic, social, and psychological well-being to their improved vision after receiving reading glasses, suggesting that the intervention had a direct impact (clarified now in LINE)

While the lack of a control group limits our ability to fully control for all confounders, we attempted to mitigate this through a pre- and post-intervention design, which allowed us to compare participants with themselves over time. We also considered potential confounding variables in our logistic regressions analysis, such as all the demographics profiles (clarified

now in LINE), though further research with a randomized design and control group would be valuable in confirming these results.

2. Introduction: No changes required.

3. Methods: Rationale for why mixed method was adopted to answer the research question can be added. However, my suggestion to the author is to split this into two manuscripts. For this manuscript the authors can handle only the quantitative data. The challenge in combined study design is clearly seen in the manuscript through lean representation of the qualitative component or information.

Response: We politely disagree with this suggestion for methodological reasons. The study is a non-controlled before-after study, which presented shortcomings to show causal relationships between spectacle correction and women's empowerment. As was pointed out earlier, the positive results could be influenced by known and unknown confounders. The approach we took to overcome this is to include a qualitative component to understand whether these associations were a direct effect from the spectacle correction. We have included the rationale in LINE 399-405.

We agree to the comment of lean representation of qualitative study as we had to write within the word limit posed by the journal guidelines and could not include the full description of the narrative analysis conducted. Please see revised Results.

4. Results and discussion: I have no comments for the quantitative data and its aligned discussion points. I would recommend the authors to rewrite the whole manuscript removing the qualitative aspect and make changes accordingly.

Response: See responses to Question 1 and 3, and comment from and response to the Editor.

Reviewer: 2

Dr. Chris Dickinson, Manchester University

Comments to the Author:

This is an interesting study which appears to follow on from earlier work by the same research group on empowerment and near vision correction (references 22 and 23).

The Introduction clearly sets out the aim of the study – to test the hypothesis of improvement in self-reported empowerment using a questionnaire, and to use focus groups to further understand and interpret these findings. Some of the sections in the Introduction are a bit cryptic, and it isn't clear what point the authors are trying to make: in particular lines 79-83 and lines 88-90.

Response: We have amended this.

It was surprising that greater use did not seem to be made of the findings of reference 23 in developing the questionnaire. Nonetheless the methodology appears reasonable, although the questionnaire was not validated. It is also not an ideal design that all questions are phrased in the same positive way, rather than having some where the "positive" response would have been to disagree with the statement.

Response: Thank you for the comment. We agree that positive phrasing may have overestimated the effects of spectacle correction. This is now clarified in Limitations (LINE401-403).

The process of provision of spectacles is not very clear. Did any of these women have uncorrected distance refractive errors and were these corrected as well? Was any payment required from the women for the spectacles (and is there a sustainable plan to continue to supply in the future?).

Response: Women with uncorrected distance refractive errors were corrected. All spectacles were provided for free (as per the ethical requirements from the Zanzibar Institutional Review Board). This is now clarified in LINE 149-150)

Women have access to governmental hospital and eye centre for spectacles in the future. The Government is currently reviewing the inclusion basic refractive care as part of the National Health Insurance Scheme. The evidence provided in this study contributes to this policy decision.

What was the approximate response rate to the initial invitation to the eye health screening – how many women were approached to participate? Were the questionnaires conducted verbally or in writing?

Response: The response rate to initial invitation of those 40 years and older and was 87.4% (209 out of 239) (now clarified in LINE 227-229). The questionnaires were conducted verbally (now clarified in LINE 182).

How were the participants for the focus group selected?

Response: The participants for the focus group were purposively selected, where it included women engaged in different craftwork (Weaving, n=5; Tailoring and sewing, n=5, Pottery=4 and Producing oil and making soaps, n=4), location (Unguja, n=10, Pemba, n=8) and Years involved in their crafts (less than 10 years, n=11, more than 10 years, n=7). (LINE195-199)

The questions asked in the focus group don't appear to relate to empowerment. The sessions are then referred to as interviews?

Response: The reviewer raised an important point on the questions. We have used indirect questioning and narrative method (overarching questions with probes on their financial situation, social life etc.) for several of reasons.

1. In a patriarchal society, the topic of empowerment is seen as a sensitive issue. Our approach allows us to navigate these cultural sensitivities and gather meaningful data without causing discomfort or misunderstanding.
2. We also acknowledge that by using direct questioning on empowerment, the craftswomen might respond in ways they believe are socially acceptable or expected from them, rather than being fully honest. Indirect questioning helps minimize this bias by allowing the craftswomen to express their thoughts more naturally, without feeling pressured to conform to perceived social norms.
3. Empowerment in also a concept that is new among older women in Zanzibar. We were concerned that it can be a complex and nuanced concept that they might not fully understand or be able to articulate directly.

We clarified this further in our Methods. (LINE 200-206)

The 30-minute duration seems very short for a focus group with facilitated discussion. Did these take place after the follow-up questionnaire?

Response: We agree that 30 minutes were a short duration. This was from the smallest group with five women. For this particular group, the women were relatively highly enthusiastic compared to the other two groups, clearly understood the questions and the topic, and feel confident in their answers and responded very quickly to the questions. Our facilitators also guided the discussions and kept it focused.

We also did not include the 15-minute ice-breaking activities in the calculation of the focus group duration. When included, the duration ranged from 45 minutes to 65 minutes. (LINE 212)

The focus group discussions were conducted after the follow-up questionnaire.

The presentation of the results of this part of the study is very disappointing. The method describes coding and developing themes, but these are not really apparent in the presentation – there just seems to be a selection of a few quotes, some of which are not particularly illustrative of the point being made (eg lines 221-222, and lines 232-234), and I don't believe they fulfil the aim of explaining the questionnaire responses. The "key messages" statement that "craftswomen also provided detailed accounts of how their lives have transformed" also rather overstates what is reported in the paper.

Response: To adhere to the journal's word limit, we kept the reporting of the focus group discussion very brief in our original submission. However, we have noted the feedback and have rewritten the narrative analysis for the qualitative component in the Results section.

In the results on Line 187 (and in the abstract) shouldn't the 157 participants be 75.1% rather than 74.1%?

Response: Thank you for pointing out the miscalculation. This is now corrected.

In the quantitative results, it is confusing that the text does not use the same wording as the questions. There are numerous examples of this: eg in the text "agreed that they were able to effectively manage their businesses and make important decisions" but the question was actually "I can run a good business".

Response: Thank you for pointing this out. We have amended this.

The Discussion does not address any issues with confounders. For example, what is meant in lines 137-140 by "promoting empowerment"? Could the women have been influenced by factors other than the spectacles?

Response: We acknowledge that, in the absence of a control group, our study's quantitative results may be influenced by confounding factors such as changes in social engagement, economic opportunities, or household dynamics unrelated to the intervention. However, we believe our mixed-method approach—combining both quantitative data and qualitative insights—helps provide a holistic view of the empowerment process (clarified now in LINE 403-405). The qualitative findings indicate that participants explicitly attributed improvements in their economic, social, and psychological well-being to their improved vision after receiving reading glasses, suggesting that the intervention is associated to the direct impact.

However, the lack of a control group limits our ability to fully control for all confounders. We attempted to mitigate this through a pre- and post-intervention design, which allowed us to compare participants with themselves over time. We also considered potential confounding variables in our logistic regressions analysis, such as all the demographics profiles (clarified now in LINE 187-189), though further research with a randomized design and control group

would be valuable in confirming these results, if deemed ethical by local IRB to have a control group in a study that withholds an intervention that is proven safe and inexpensive until the end of trial.

Would they have wanted to be positive about the effect of spectacles to please the researchers?

Response: We agreed with the reviewer, and hence we included a qualitative component that used indirect questioning to minimise the bias by encouraging more natural responses, as direct questioning might lead craftswomen to provide socially acceptable answers (clarified in LINE401-403)

It would also be worth discussing that many aspects of empowerment also appeared high before the intervention – typically 80-90% of the responses were agree or strongly agree, even at baseline. Therefore, even if the results are statistically significant, are they actually practically significant in changing levels of empowerment? The limitations of the questionnaire design should also be addressed.

Response: We recognize that many participants reported high levels of empowerment before the intervention. To assess the relative improvement in responses, we calculated odds ratios for each statement, focusing on the likelihood of upward movement on the Likert scale (clarified in LINE187-189). This approach differs from calculating proportion of 'Agree' and 'Strongly Agree' responses post intervention. We have also discussed the limitation of the questionnaire in LINE 401-403).

The authors should take care not to go too far into speculation in their Discussion (and in the conclusion of the Abstract). Its not clear what evidence they have for needing a longer timeframe to assess results. They should also be careful in their comments hinting at entrepreneurship and mentoring, since three questions on this issue (and a couple more in the "education" category which are related) were removed from the final questionnaire.

Response: Thank you for the comment. The research team's view is that while a six-month follow-up can provide valuable insights, it might not be sufficient to fully understand the long-term impact of spectacle correction on the empowerment of craftswomen in Zanzibar. We suggested a longer timeframe to study empowerment for certain reasons, as discussed by a empirical analysis in Bangladesh experience (Kabeer N, Mahmud S, Tasneem S. The contested relationship between paid work and women's empowerment: Empirical analysis from Bangladesh. The European Journal of Development Research. 2018 Apr;30:235-51.) and insightful reflection by Kabeer N. (Resources, agency, achievements: Reflections on the measurement of women's empowerment. Development and change. 1999 Jul;30(3):435-64).

1. Empowerment is a complex process that often involves gradual shifts in social roles, economic opportunities, and self-perception. These changes may take more than six months to materialize.
2. Overcoming deeply ingrained cultural norms and stereotypes can be a lengthy process.
3. Measuring empowerment is a multifaceted task. It often involves assessing changes in self-esteem, decision-making power, economic independence, and social status. A six-month follow-up might not capture the full extent of these changes.

We agreed that it might be premature to recommend a solution via entrepreneurship mentoring and omitted this in both the abstract and the discussion by removing the comment on the need for systematic business mentoring.

Reviewer: 1
Dr. Majid Moshirfar, University of Utah
Comments to the Author:
1. It is disappointing to see that there was a significant loss of individuals to follow-up. For better transparency of your study, I think it is important that in the abstract, you reflect only 157 patients with follow-up. There is no point in mentioning that there were 209 participants when in reality, you analyzed 157 individuals. Therefore, I think you should remove the word “209 participants” from your abstract for better transparency.

Response: Thank you for pointing out the high loss of follow up. We have removed the word “209 participants” from our abstract for better transparency, as suggested.

2. Realizing that the level of presbyopia could have been different among your age group, is there any way you can provide us the range, distribution of presbyopia for this study, perhaps from +1.25 to +3.00 based on the age?

Response: Yes. We have not included this information in the manuscript because we did not aim to conduct multiple subgroup analyses. The small sample size in the cells can lead to Type I error (false positives) or reduce the apparent significance of true effects, making it difficult to test for significance.

**Prescription of near reading glasses (Dioptre)**	**40-50 years N, row %**	**51-60 years N, row %**	**>60 years N, row %**
+1.00D to +1.75D	43, 93.5%	2, 4.3%	1, 2.2%
+2.00D to +2.75D	31, 42.1%	35, 47.4%	8, 10.5%
> +3.00D	5, 13.5%	26, 70.3%	6, 16.2%

3. Is there any way you can correlate the level of presbyopia or severity of presbyopia with the improvement in patient economics, social, and psychological empowerment based on the severity of presbyopia after six months? In other words, can you do any correlation between the severity of presbyopia and the improvement in empowerment over time?
Response: Yes. We have performed logistic regression on all demographic profiles including the level of presbyopia, but found no statistical association difference after correction in all domains of empowerment (p>0.05). As mentioned above, we hypothsise that this could be an issue for sub-group analyses where small cell sizes contribute to a lower overall sample size, reducing the statistical power of the test.

4. The authors are quite correct that there may be a cultural factor, which is why political empowerment may not be reflected in such a short amount of time.
Response: Thank you.

5. Are there any adverse outcomes that the authors can reflect in this manuscript concerning some of the surprising detrimental effects as a result of improvement and gain of confidence among this group of patients?
Response: From the qualitative focus group discussions in the current study, the craftswomen have not mentioned adverse outcomes as a result of improvement and gain of confidence. However, there were two subsequent studies that

explored this in-depth through: 1. interviews with the craftswomen's husbands and community leaders, 2. solicited diaries from 10 craftswomen documenting their experiences and 3. a follow-up workshop with 20 craftswomen. The data from these three `studies are being analysed currently and will be published separately.

6. Is there any way you can provide the power of your study based on your sample size?
Response: Based on our final sample size of 157, with a Type II error rate, α (two-tailed) = 5.0%, estimated effect size, EE=0.15, a standard deviation and SΔ = 0.5, the power of the study reached 95%. We clarified this in LINE 158-160.

7. I congratulate the authors for their interesting work. I think this is a nicely conducted study that is worthy of publication.
Response: Thank you.

Reviewer: 2
Prof. Charles O. Bekibele, University of Ibadan
Comments to the Author:
Good study, relatively high loss to follow up but understandable in an African setting where loss to follow up is often very high.

Response: Thank you.

VERSION 2 - REVIEW

**Reviewer**	**2**
**Name**	**Dickinson, Chris**
**Affiliation**	**Manchester University**
**Date**	**07-Oct-2024**
**COI**	

Thank you to the authors for their positive response to my comments, and for the clarifications they have added to the paper with strengthens their message.

I do still have a few minor issues which I feel could be addressed.

From the paper:

Abstract Results section is slightly confusing. It starts by talking about quantitative results, then qualitative and then quantitative – move the sentence about qualitative findings to the end.

Lines 325-335 As I wrote in my initial review, I do not believe that these quotes support the interpretations presented – could you substitute for something more relevant?

Lines 410-412 I don't think the added bit on psychological empowerment is appropriate – the changes in this are the least of all those reported. Suggest deleting the new sentence.

Lines 447-450 It would be better to delete the final sentence, and end with "…social and psychological well-being".

From the Authors' Response:

You say the evidence provided in this study is being used to inform Government policy. It would be good to include this as evidence of impact, maybe as part of "strengths and limitations".

In my earlier review I queried the "real life" significance of the changes in empowerment when 80-90% of responses were agree or strongly agree at baseline. I understand that the Odds Ratios do indicate STATISTICAL significance but that is not the same as showing that there is a positive effect in everyday life. The authors could counter with the argument that their qualitative work shows that there is a real effect, but I think this needs to be explicitly addressed in the Discussion.

Minor points

Line 52 "…understanding their capabilities.."

Line 61 "..design provides…"

Line 66 Would be better to say "Self-reported and positive phrasing questionnaire in this study…."

Lines 105 -108 "..with women facing….and having 7.8%...". ….in low income countries face in different aspects of their lives….."

Lines 267-270 This sentence makes more sense with "Although.." added back in

Line 320 Missing closing bracket

Line 348-349 The text is presented in italics which is usually reserved only for the quotes

Lines 372-373 These are 2 separate quotes. Please present them in that way.

VERSION 2 - AUTHOR RESPONSE

Reviewer: 2
Dr. Chris Dickinson, Manchester University
Comments to the Author:
Thank you to the authors for their positive response to my comments, and for the clarifications they have added to the paper with strengthens their message.

I do still have a few minor issues which I feel could be addressed.

From the paper:
Abstract Results section is slightly confusing. It starts by talking about quantitative results, then qualitative and then quantitative – move the sentence about qualitative findings to the end.
Response: Amended.

Lines 325-335 As I wrote in my initial review, I do not believe that these quotes support the interpretations presented – could you substitute for something more relevant?
Response: Thank you for the suggestions. We have reviewed our code book and included more relevant quotes to demonstrate psychological empowerment. (Lines 317 to 326)

Lines 410-412 I don't think the added bit on psychological empowerment is appropriate – the changes in this are the least of all those reported. Suggest deleting the new sentence.
Response: Amended.

Lines 447-450 It would be better to delete the final sentence, and end with "…social and psychological well-being".
Response: Amended.

From the Authors' Response:
You say the evidence provided in this study is being used to inform Government policy. It would be good to include this as evidence of impact, maybe as part of "strengths and limitations".
Response: Added to Strengths and Limitation.

In my earlier review I queried the "real life" significance of the changes in empowerment when 80-90% of responses were agree or strongly agree at baseline. I understand that the Odds Ratios do indicate STATISTICAL significance but that is not the same as showing that there is a positive effect in everyday life. The authors could counter with the argument that their qualitative work shows that there is a real effect, but I think this needs to be explicitly addressed in the Discussion.

Response: Thank you for your insightful comment regarding the “real-life” significance of the changes in empowerment. We acknowledge the importance of distinguishing between statistical significance and practical, everyday significance of the findings.

While the Odds Ratios indeed demonstrate statistical significance, we agree that this does not automatically imply that the observed changes are meaningful in participants' everyday lives. To address this, we have revised the Discussion section to explicitly highlight how the qualitative data supports the interpretation that these changes in empowerment, though statistically significant, also reflect tangible, positive effects in the daily lives of the participants.

Specifically, the qualitative work revealed detailed accounts of how vision correction enabled the participants to improve their productivity, income generation, and self-confidence in their crafts. These narratives provide rich context and reinforce that the changes observed are not just numerical but are experienced as meaningful improvements by the participants.

We have included a discussion of how these qualitative insights complement the quantitative findings, making a clearer case that the changes in empowerment go beyond statistical metrics and have practical implications in their social and economic empowerment. (Lines 408 to 415)

Minor points
Line 52 “…understanding their capabilities..”
Response: Amended.

Line 61 “..design provides…”
Response: Amended.

Line 66 Would be better to say “Self-reported and positive phrasing questionnaire in this study….”
Response: Amended.

Lines 105 -108 “..with women facing….and having 7.8%...”. ….in low income countries face in different aspects of their lives…..”
Response: Amended.

Lines 267-270 This sentence makes more sense with “Although..” added back in
Response: Amended.

Line 320 Missing closing bracket
Response: Amended.

Line 348-349 The text is presented in italics which is usually reserved only for the quotes

Response: Amended.

Lines 372-373 These are 2 separate quotes. Please present them in that way.
Response: Amended.
